# Rapid laser solver for the phase retrieval problem

**DOI:** 10.1126/sciadv.aax4530

**Published:** 2019-10-04

**Authors:** C. Tradonsky, I. Gershenzon, V. Pal, R. Chriki, A. A. Friesem, O. Raz, N. Davidson

**Affiliations:** 1 Department of Physics of Complex Systems, Weizmann Institute of Science, Rehovot 7610001, Israel.; 2 Department of Physics, Indian Institute of Technology Ropar, Rupnagar 140001, Punjab, India.

## Abstract

Tailored physical systems were recently exploited to rapidly solve hard computational challenges, such as spin simulators, combinatorial optimization, and focusing through scattering media. Here, we address the phase retrieval problem where an object is reconstructed from its scattered intensity distribution. This is a key problem in many applications, ranging from x-ray imaging to astrophysics, and currently, it lacks efficient direct reconstruction methods: The widely used indirect iterative algorithms are inherently slow. We present an optical approach based on a digital degenerate cavity laser, whose most probable lasing mode rapidly and efficiently reconstructs the object. Our experimental results suggest that the gain competition between the many lasing modes acts as a highly parallel computer that could rapidly solve the phase retrieval problem. Our approach applies to most two-dimensional objects with known compact support, including complex-valued objects, and can be generalized to imaging through scattering media and other hard computational tasks.

## Teaser

An optical system solves the phase retrieval problem by exploiting the natural mode competition inside a degenerate laser cavity.

## INTRODUCTION

Calculating the intensity distribution of light scattered far from a known object is relatively easy: It is the square of the absolute value of the object’s Fourier transform (*1*). However, reconstructing an object from its scattered intensity distribution is generally an ill-posed problem because the phase information is lost and different choices of phase distributions result in different reconstructions. Fortunately, in many applications, additional prior information—e.g., the object’s shape, positivity, spatial symmetry, or sparsity—can be exploited to remove extraneous phase distributions and, hence, retrieve the original phase distribution and enable object reconstruction. Examples for applications can be found in astronomy (*2*), short-pulse characterization (*3*), x-ray diffraction (*4*), radar detection (*5*), speech recognition (*6*), and imaging through turbid media (*7*, *8*).

For objects with a finite extent (compact support), a unique solution to the phase retrieval problem almost always exists (up to trivial ambiguities), provided that the scattered intensity is sampled at a sufficiently high resolution (*9*). During the last decades, several algorithms for solving the phase retrieval problem have been developed. These include the Gerchberg-Saxton (GS) error reduction algorithm (*10*), hybrid input-output algorithm (*11*), relaxed averaged alternating reflections (RAAR) algorithm (*12*), difference map algorithm (*13*), and shrink-wrap algorithm (*14*) [see (*15*–*17*) for a modern review]. Unfortunately, these algorithms are based on iterative projections and hence are relatively slow even with high-performance computers (*16*).

An alternative approach to address computational challenges, such as phase retrieval and other optimization problems, is through specifically tailored physical systems. These systems are not universal Turing machines, namely, they cannot perform arbitrary calculations, but they could, potentially, solve a specific class of problems very efficiently. Prominent examples for these systems are the d-wave machine that finds the ground state of a complicated Hamiltonian through quantum annealing (*18*–*20*), coupled lasers, degenerate optical parametric oscillators (OPO), and coupled polariton systems for solving various difficult optimization problems (*21*–*28*). Solving hard problems with these systems could, potentially, prove advantageous in computation time and resources over conventional computers (*21*, *29*).

Here, we present and experimentally demonstrate a novel optical system that can rapidly solve phase retrieval problems. It is based on a digital degenerate cavity laser (DDCL) (*30*, *31*), in which two constraints, the Fourier magnitudes of the scattered light from an object and the compact support, are incorporated. The nonlinear lasing process in this cavity results in a self-consistent solution that best satisfies both constraints. An upper bound of 100 ns was measured for the time needed by the DDCL to converge to a stable solution.

The physical mechanism that generates the nonlinear lasing process in DDCLs is similar to that of the OPO spin simulators (*23*, *24*, *26*). Both the OPO simulators and the DDCLs have distinct advantages in performing optimization, including extremely fast operation (*21*, *26*), ability to avoid local minima when the pumping is increased slowly enough (*23*, *25*), and having a non-Gaussian wave packet (*32*). The two fundamental physical processes in the system, which allow the DDCL system to find the optimal solution, are the phase locking of the lasers and the nonlinear mode competition in the cavity (*33*–*35*). The compact support aperture inside the cavity ensures that different configurations of laser phases result in different losses and the configuration with the minimal losses wins the mode competition; thereby, the solution for the phase problem is found.

Our DDCL system has several particular attractive and important features. These include high parallelism that simultaneously provides millions of parallel realizations; short round trip times (~20 ns), leading to fast convergence time; and inherent selection of the mode with minimal loss as a result of mode competition.

## RESULTS

### Experimental arrangement

**Fig. 1:**
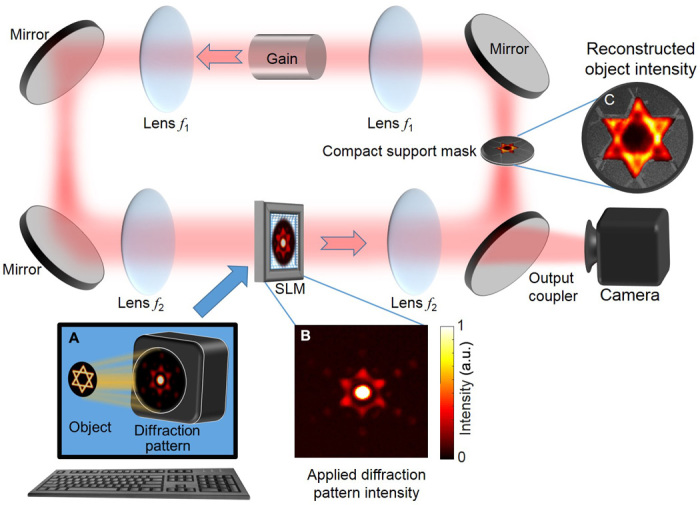
Basic DDCL arrangement for rapid phase retrieval. (**A**) Calculated scattered intensity distribution from the object (essentially the Fourier intensity distribution) is applied onto an SLM, which is incorporated into a ring degenerate cavity laser that can support up to 100,000 degenerate transverse modes. A mask shaped as the object boundaries (compact support) at the Fourier plane filters out extraneous modes that do not match the compact support. With this laser arrangement, the lasing process yields a self-consistent solution that satisfies both the scattered intensity distribution shown in (**B**) and the compact support constraint. (**C**) The reconstructed object intensity appears at the compact support mask and is imaged onto the camera. a.u., arbitrary units.

The basic DDCL arrangement for rapidly solving the phase retrieval problem is schematically presented in Fig. 1. It consists of a ring degenerate cavity laser that includes a gain medium, two 4*f* telescopes, an amplitude spatial light modulator (SLM), an intracavity aperture, three high-reflectivity mirrors, and an output coupler. The left 4*f* telescope (*f*_1_ and *f*_2_) images the center of the gain medium onto the SLM, where the transmittance at each pixel is controlled independently (*30*). The intracavity aperture, together with the SLM, serves to control and form the output lasing intensity distribution (*36*). In the absence of the intracavity aperture, the right 4*f* telescope simply reimages the SLM back onto the gain medium, so all phase distributions can lase with equal probability, i.e., the amplification and losses are phase independent. However, when an intracavity aperture (compact support mask) is placed at the Fourier plane between the two lenses, each phase distribution has a different level of loss. In this case, the phase distribution that experiences the minimal loss is the most probable lasing mode, as a result of mode competition.

Theoretically, two constraints are required to solve phase retrieval problems with our system: (i) an SLM transmittance pattern that is determined by the intensity distribution of the Fourier transform of the object and (ii) an intracavity aperture having the size and shape of the compact support. The transmittance of each SLM pixel is set to a local function of the Fourier intensity described in the theory section (Eq. 3). Because of laser cavity imperfections, we set the transmittance of the SLM using an iterative scheme outlined in section S1. The intracavity aperture size and shape, enforcing the compact support constraint, were predetermined in accordance to the known object size and shape. With both constraints of the phase retrieval problem implemented in the cavity, the most probable lasing mode corresponds to the optimal solution of the phase retrieval problem. The field distribution of any other lasing mode spreads beyond the boundaries of the intracavity aperture and therefore suffers additional loss. The field distribution of the resulting lasing mode, i.e., of the reconstructed object, is formed within the intracavity aperture and is imaged through the output coupler onto the camera. In a typical application, the Fourier intensity distribution and the compact support would both be provided as external inputs, for example, those obtained by x-ray diffraction experiments. All the experimental results reported in this work were obtained with synthetic inputs generated in the following manner. Starting from a digitized object field, the Fourier intensity distribution was calculated by a discrete Fourier transformation and then used to set the SLM transmittance. The compact support size and shape, commonly obtained by a low-resolution imaging of the object, were determined from the digitized object. The actual and detailed experimental arrangement that was used in our experiments is presented in fig. S1.

### Experimental results

We conducted extensive investigations on the performance of experimental system shown in fig. S1. We considered two figures of merit to quantify the quality of our system: solution fidelity and the computation time. The fidelity of a solution intensity distribution is calculated as

$$\text{Fidelity}=1-\frac{1}{2}{\left\|I_{\text{det}}(x)-I_{\text{orig}}(x)\right\|}_{2}^{2}$$ (1)

where *I*_det_ is the aligned normalized intensity of the detected reconstructed object (or Fourier distribution), *I*_orig_ is the normalized intensity of the original object (or calculated Fourier distribution), and ‖*I*(*x*)‖_2_ is the L_2_ norm of an intensity pattern *I*(*x*). The use of the intensity-based fidelity metric in Eq. 1 is justified by the uniqueness property of the phase retrieval problem solution (*9*). Specifically, the uniqueness property guarantees that the object phase distribution can be easily obtained from the reconstructed intensity (*10*). This is verified in section S5 and fig. S6. We also show, in section S5 and fig. S6, that the Fourier phase distribution can be directly measured in our system.

**Fig. 2:**
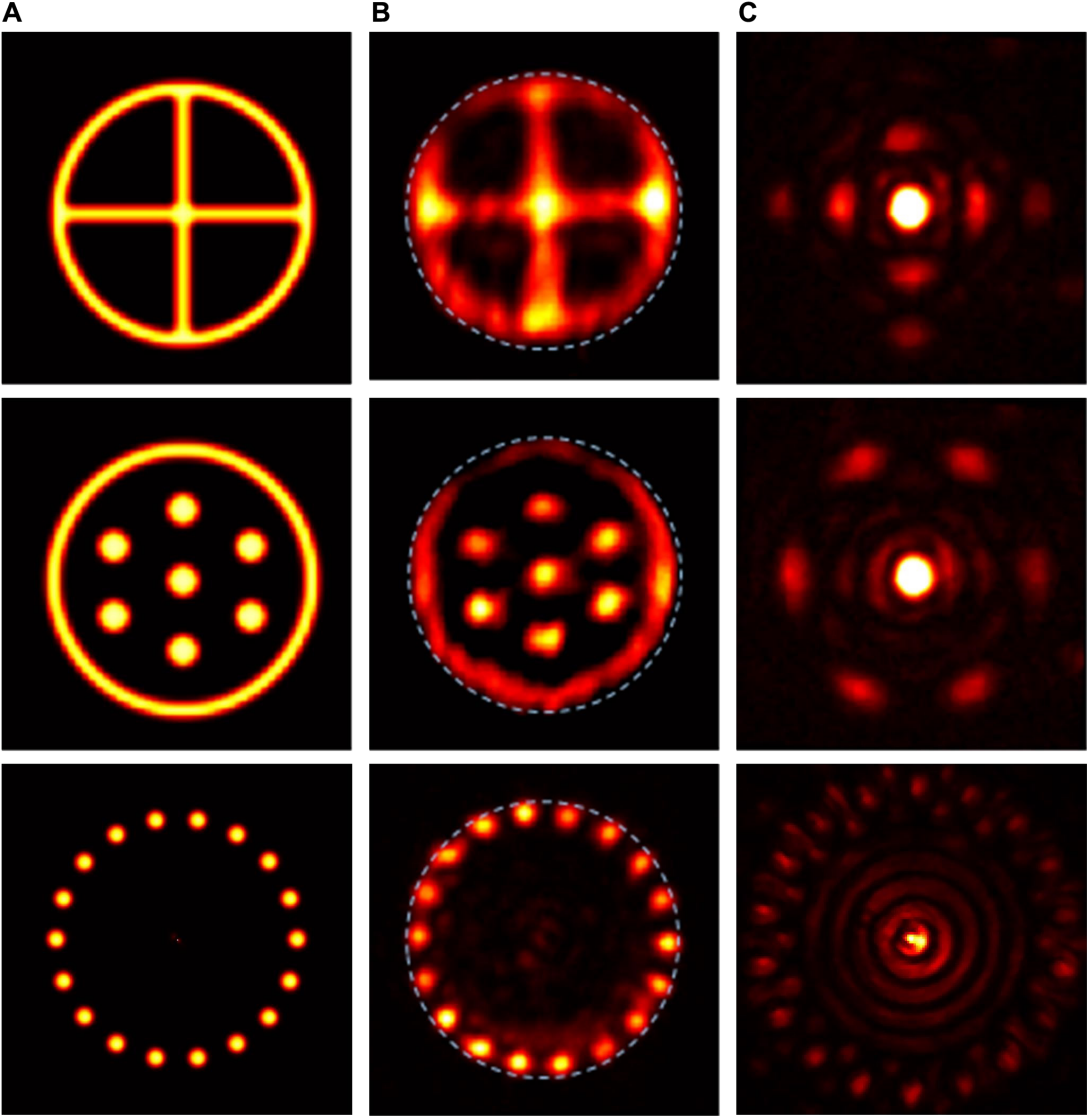
Experimental results for real-valued centrosymmetric objects. Column (**A**) Intensity distributions of the actual objects. Column (**B**) Detected intensity distribution of the reconstructed objects, using a circular aperture as compact support. Column (**C**) Fourier intensity distributions at the SLM.

**Fig. 3:**
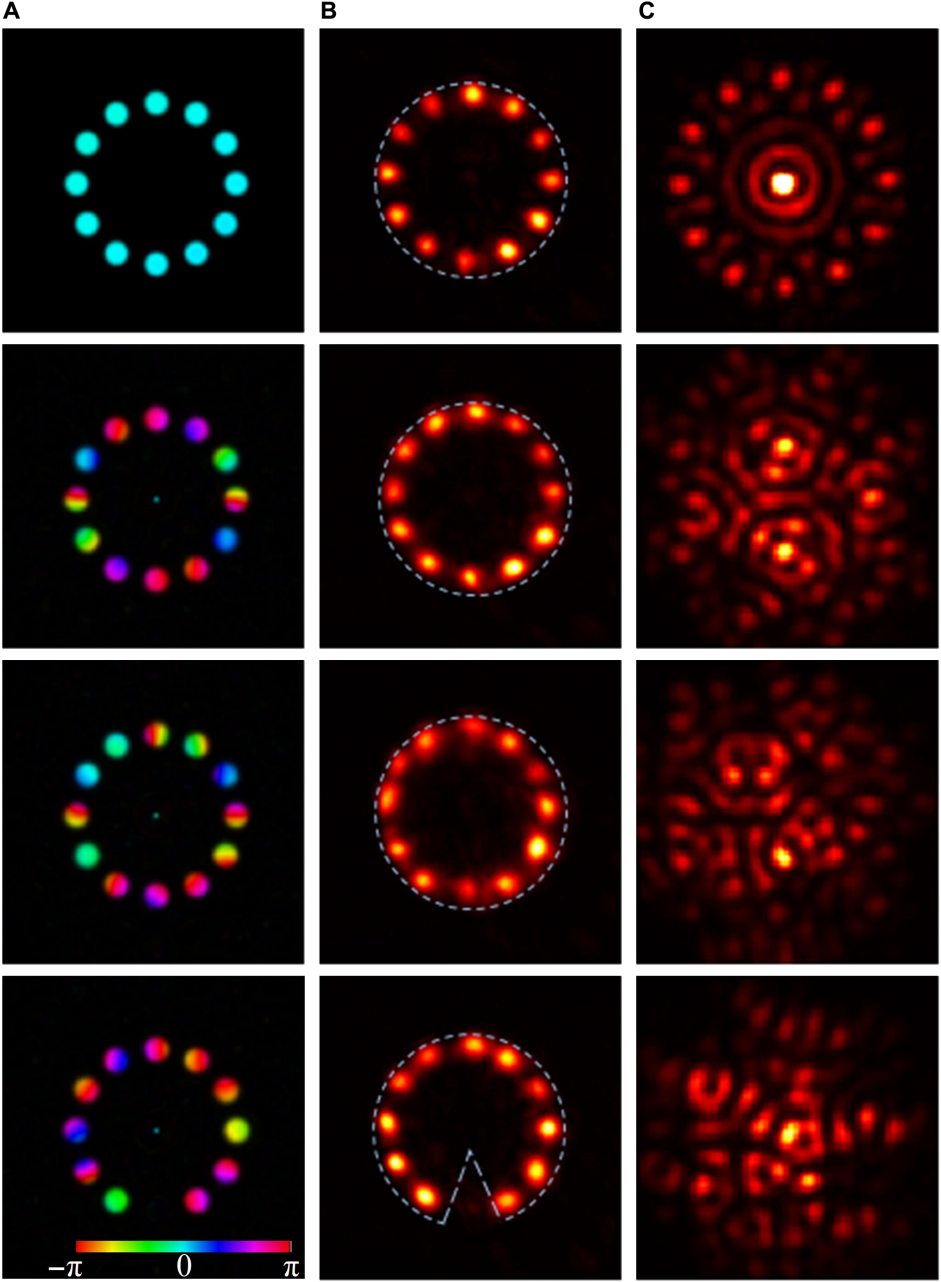
Experimental results for complex-valued objects. Column (**A**) Intensity (brightness) and phase (hue) distributions of the actual objects. Column (**B**) Detected intensity distribution of the reconstructed objects, using mainly a circular aperture as compact support. Column (**C**) Fourier intensity distributions at the SLM. The first row shows an object with uniform phase distribution with a reconstruction fidelity of 0.91. The second (third) row shows the same object with arbitrary centrosymmetric (asymmetric) phase distribution with a reconstruction fidelity of 0.89 (0.81). The fourth row shows a noncentrosymmetric object with random asymmetric phase distribution and noncircular compact support with a reconstruction fidelity of 0.91.

**Fig. 4:**
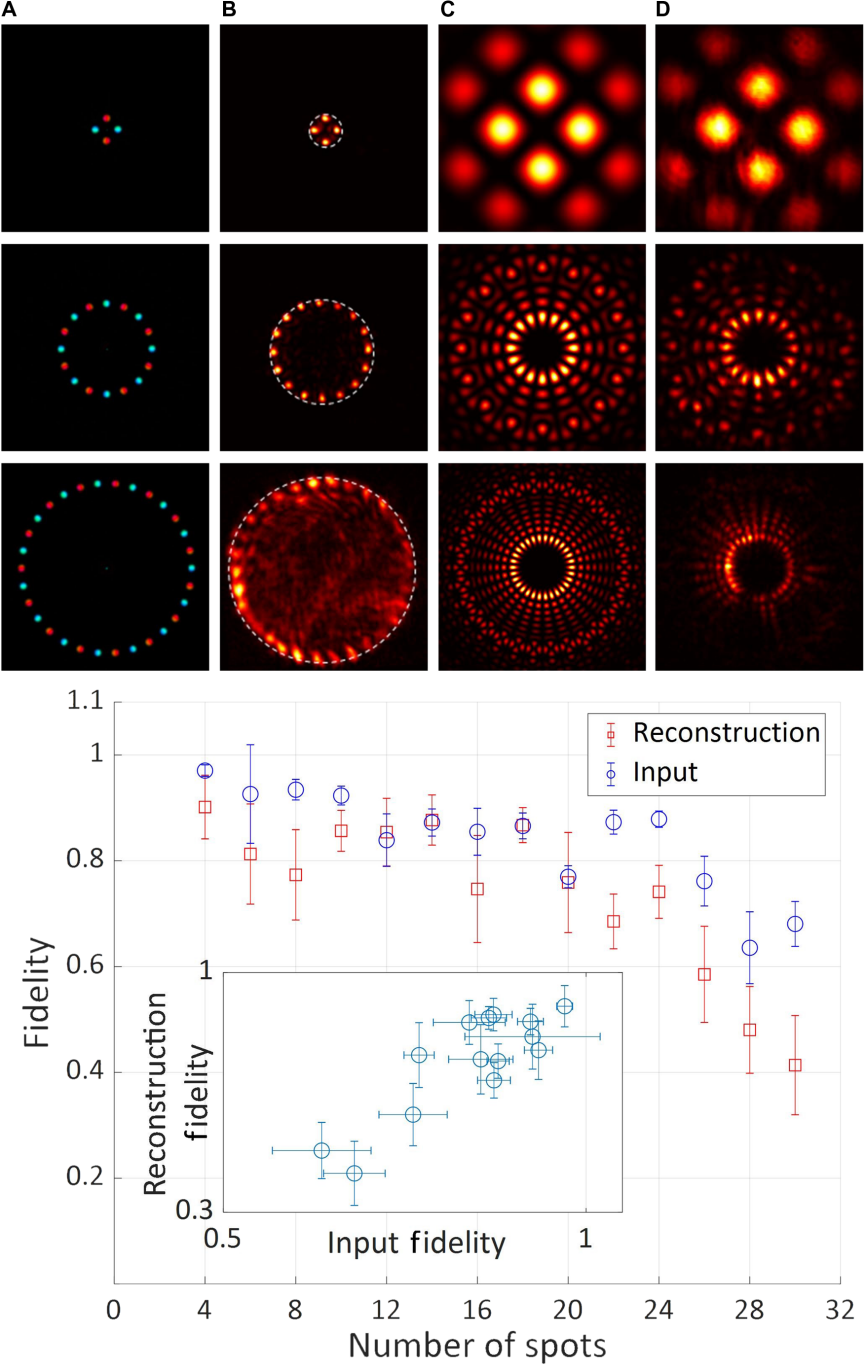
Experimental and quantitative results for fidelity as a function of object complexity. Top: Representative intensity distributions of objects with 4, 16, and 30 spots. Column (**A**) Intensity (brightness) and phase (hue) distributions of the actual objects. Column (**B**) Detected intensity distribution of the reconstructed objects, using a circular aperture as compact support. Column (**C**) Calculated Fourier intensity distributions applied to control the SLM. Column (**D**) Detected corresponding Fourier intensity distributions after modifications by SLM properties. Bottom: Quantitative fidelity values of the Fourier intensity distributions (blue) and the reconstructed object intensity distributions (red) as a function of the number of spots in the object (4 to 30). Inset: Fidelity values of the reconstructed object intensity distributions as a function of the fidelity values of the Fourier intensity distributions for all the measurements.

**Fig. 5:**
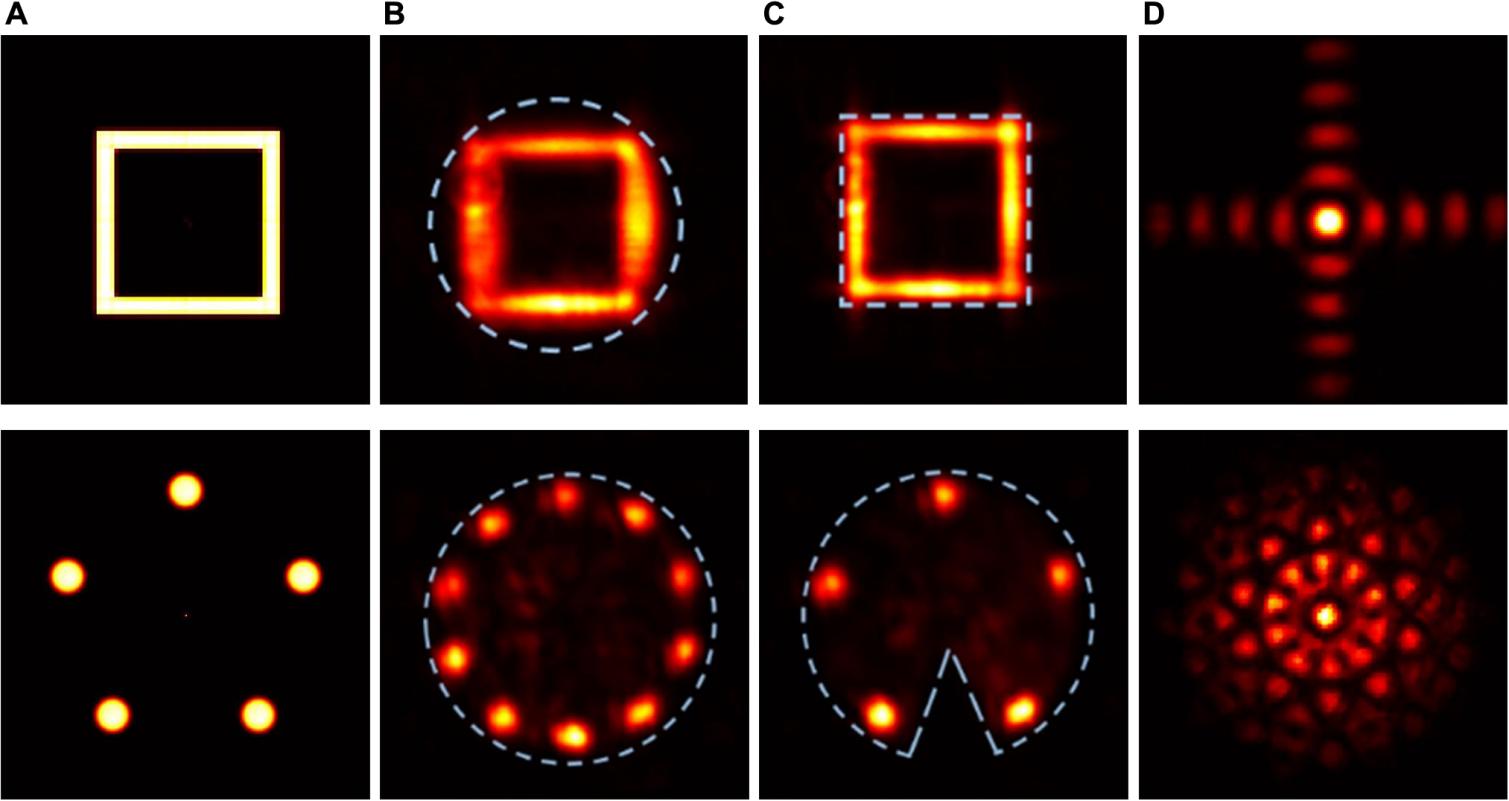
Experimental results demonstrating the qualitative effect of tightness and asymmetry of compact supports. Column (**A**) Intensity distribution of the actual objects. Column (**B**) Detected intensity distribution of the reconstructed objects, using a circular aperture as compact support. Column (**C**) Detected intensity distribution of the reconstructed objects, using a square aperture as tight compact support (top row) and a circular aperture with a wedge as asymmetric compact support (bottom row). Column (**D**) Fourier intensity distributions at the SLM.

Representative experimental results, including the fidelity figures of merit, for different objects are presented in Figs. 2 to 5. In these figures, columns A show the intensity and/or phase distributions of the actual objects; columns B (and column C in Fig. 5) show the detected intensity distributions of the reconstructed objects along with the compact support outlines; columns C (column D in Figs. 4 and 5) show the intensity distributions at the SLM (calculated Fourier intensity distributions of the object after modification by the SLM properties), which controlled the transmission of the SLM (see section S1); and column C in Fig. 4 shows examples of calculated Fourier intensity distributions before modification by the SLM properties.

Representative results for centrosymmetric objects with uniform phase distribution (i.e., real-valued objects) and circular compact support are presented in Fig. 2. As evident, there is very good agreement between the intensity distributions of the actual objects and those of the reconstructed objects. Figure 3 shows the results for objects with centrosymmetric intensity distributions and various complex phase distributions and circular compact support. The first row shows the results for an object with uniform phase distribution, where the corresponding Fourier intensity distribution has a 12-fold symmetry. The second row shows an object with centrosymmetric phase distribution, so both the object and the corresponding Fourier intensity distribution are centrosymmetric. Note that our system correctly reconstructs the actual objects, although the Fourier intensity distributions for the first and second rows are notably different. The high-quality reconstructions (reconstruction fidelities of 0.81 to 0.91) indicate that our approach is also valid for complex-valued objects, which are generally harder to solve computationally (*37*).

A known ambiguity in phase retrieval emerges when the object is noncentrosymmetric, but the assumed compact support is centrosymmetric. An example for such a case is shown in the third row of Fig. 3, where the object has a random asymmetric phase distribution so the corresponding Fourier intensity distribution is also asymmetric. Here, the reconstruction fidelity is only 0.81, and the blurred reconstructed object differs from the actual object because of interferences between two degenerate solutions (one is the image of the object and the other the inverted phase-conjugated image). Applying a properly designed, noncentrosymmetric compact support (for a noncentrosymmetric object) removes this degeneracy, so as to obtain a high-quality reconstruction with a fidelity of 0.91, as shown in the fourth row of Fig. 3. Note that the design of the noncentrosymmetric compact support requires knowledge about the asymmetry of the object. For example, in x-ray applications, this can usually be obtained by low-resolution imaging of the object.

The effect of object complexity on the reconstruction fidelity was measured and is presented in Fig. 4. The original objects consisted of an array with an even number of spots and alternating phases of 0 and π, arranged in a ring geometry. The number of spots ranged from 4 to 30, while the size of all spots was kept constant. Each data point in Fig. 4 is made up of the average fidelities of the input Fourier and reconstructed object intensity distributions obtained in 48 repetitions for each number of spots. Representative intensity distributions for objects with 4, 16, and 30 spots are presented in the top part of Fig. 4. These indicate that higher-complexity objects (with more spots) have a higher-complexity Fourier intensity distribution with smaller details that cannot be properly resolved by our system. The quantitative experimental results for the fidelity as a function of object complexity (number of spots) are shown in the bottom part of Fig. 4. As expected, both the input and reconstruction fidelities decrease as the object complexity increases. In addition, these fidelities are highly correlated (Pearson correlation value of 0.76; see inset of Fig. 4). The fidelity correlation results suggest that improving the fidelity between the calculated and measured input Fourier intensity distributions will improve the fidelity of the reconstructed object. Note that both fidelities exhibited substantial statistical spreading, quantified by error bars, around their mean values. These fluctuations are caused by technical noise arising from the laser pumping method (flash lamp), which could be alleviated by resorting to diode pumping.

The experimental results on the qualitative effect of tightness and symmetry of the compact support on the reconstruction quality are presented in Fig. 5. Column A shows the intensity distributions of the actual objects; columns B and C show the detected intensity distributions of the reconstructed objects along with the compact support outlines, and column D shows the intensity distributions at the SLM, which controlled the transmission of the SLM. The results in the first row demonstrate that a tight compact support (square rather than a circular aperture) substantially improves the quality of reconstructed square object. However, since both the object and the support are centrosymmetric, even the nontight circular aperture leads to a reasonable reconstruction. The results in the second row demonstrate the importance of centrosymmetry, as also shown in Fig. 3.

**Fig. 6:**
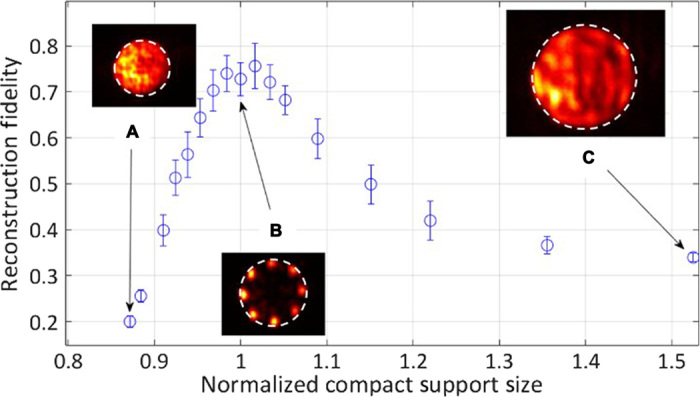
Experimental quantitative results for reconstruction fidelity as a function of the compact support radius of the aperture normalized by the object size. Insets: Typical reconstructed object intensity distributions. (**A**) Compact support radius is 87% of the object radius. (**B**) Object radius is equal to compact support radius. (**C**) Compact support radius is 152% of the object radius.

The quantitative effect of tightness of the size of the compact support on the reconstruction quality and fidelity is presented in Fig. 6. It shows the reconstruction fidelity as a function of the radius of the compact support aperture normalized by the object size and representative intensity distributions in insets A to C. As expected, when the object size is bigger than the compact support, there is a rapid decay in the reconstruction fidelity since the laser cannot support the correct object shape. When the object size is smaller than the compact support aperture, there is a slower decay in the fidelity due to overlap of multiple solutions that are supported by the aperture. Specifically, when the compact support is larger than the reconstructed object size, many translated solutions, known to be “trivial ambiguities,” are valid. Accordingly, the reconstruction fidelity is reduced when the camera averages over multiple realizations, as in our system.

Typically, the resolution of the reconstructed objects was relatively low (about 20 × 20, corresponding to a spot size of 500 μm). As indicated by numerical simulations (see section S3 and fig. S4), the resolution is limited mainly because of phase aberrations in the laser cavity. We believe that reducing these aberrations, e.g., by proper compensation with the intracavity SLM, could improve the resolution. Complete elimination of phase aberrations should substantially enhance the resolution to that of the SLM, which, in our system, would be 500 × 500 (corresponding to a spot size of 20 μm). An additional limiting factor on the reconstruction fidelity is the dynamic range of the input Fourier intensity distribution, which is often very high, e.g., in x-ray applications. In the current system, the dynamic range is quite limited. As already visible in Fig. 2, objects exhibiting a bright central peak in the Fourier distribution (the two upper panels in Fig. 2) tend to have poorer reconstruction fidelity compared to objects where the central peak is dimmer (bottom panels in Fig. 2). The dynamic range could be improved by controlling the spatial distribution of the pump intensity (end pumping).

Last, we analyze the time to solution of the system. In our system, two different processes contribute to the overall computation time. The first process includes the overhead durations for forming the specific intensity pattern on the SLM and for detecting the solution with a camera. These durations are dictated by the SLM response time and the complementary metal-oxide semiconductor camera readout time and, together, are about 20 ms. The second important process is the actual computation time of lasing, which, as discussed below and in fig. S2, is less than 100 ns. Unlike most other computational devices, where the input/output overhead duration is negligible compared to the computation time, in our system, it is the bottleneck in the total computation time. Fortunately, this bottleneck can be alleviated (reducing overhead durations to submilliseconds) by resorting to improved input/output devices, which are continuously becoming available. To determine an upper bound on the minimum duration needed for the laser to find the optimal lasing mode and reconstruct the object, we resorted to a Q-switched linear degenerate cavity laser arrangement that included a Pockels cell, as shown in fig. S2. The shortest pulse that we could generate was 100 ns long, as shown in the pulse profile in fig. S2B, and even then, the laser reconstructed the object successfully. In general, the results obtained with Q-switching were essentially the same as those with quasi–continuous wave (CW) lasing operation. This indicates that the computation time of the system is, at most, 100 ns. For comparison, we measured the median reconstruction time of the RAAR algorithm on the problem shown in the bottommost panels of Fig. 3 and found it to be about 1 s (see section S4 for additional details).

### Theory

The lasing mode in the system is a complex field at the SLM, $E(\vec{k},t)$, where $\vec{k}$ is the position at the SLM plane, mapped onto itself after propagating through the cavity. In other words, it is a stationary solution of the field propagation equation, which, for a cavity round trip of duration τ, can be written as

E(k→,t+τ)=e−αTSLM(k→)G(k→,t)F−1[M(x→)F[E(k→,t)]] (2)

where $T_{\text{SLM}}(\vec{k})$is the amplitude transmittances at the SLM; $G(\vec{k},t)$ is the (nonlinear) gain of the system; α is the loss coefficient per round trip; F is a two-dimensional Fourier transform (performed by the lenses); and $M(\vec{x})$ represents the spatial compact support imposed by the intracavity mask, where $\vec{x}$ is the position at the mask plane. Note that the mapping of $E(\vec{k},t)$ is nonlinear because of the nonlinear gain with saturation (*38*) $G(\vec{k},t)=\text{exp}(g_{0}{\left(1+{|E(\vec{k},t)|}^{2}/I_{\text{sat}}\right)}^{-1})$, where *g*_0_ is the linear gain at very low intensities set by the pumping strength and *I*_sat_ is the saturation intensity.

Now, consider the electric field $E_{\text{sol}}(\vec{k})$, which corresponds to the solution for the phase retrieval problem. This field passes through the compact support without any changes, $F^{-1}[M(\vec{x})F[E_{\text{sol}}(\vec{k})]]=E_{\text{sol}}(\vec{k})$. Assuming that $E_{\text{sol}}(\vec{k})$ is a stable, time-independent solution of Eq. 2, $e^{-\alpha }T_{\text{SLM}}(\vec{k})G_{\text{sol}}(\vec{k})=1$. With this

TSLM(k→)=exp(α−g0(1+∣Esol(k→)∣2/Isat)−1) (3)

the solution of the phase retrieval is a possible lasing mode in the system. Note that the SLM transmittance, *T*_SLM_, is a local function of the Fourier intensity ${|E_{\text{sol}}(\vec{k})|}^{2}$ and cavity parameters. Substituting the expressions for $T_{\text{SLM}}(\vec{k})$ and $G(\vec{k},t)$ into Eq. 2 yields

E(k→,t+τ)=exp(g0(11+∣E(k→,t)∣2/Isat−11+∣Esol(k→)∣2/Isat))F−1[M(x→)F[E(k→,t)]] (4)

Equation 4 can be considered as a modified GS iterative projection process, in which the fastest growing mode corresponds to the solution. To verify our approach, we performed numerical simulations of the cavity. The simulation results are presented in fig. S3, along with additional details on the numerical simulations.

At the transition to lasing, the amplified spontaneous emission (ASE) modes with the highest energy win the mode competition over the limited gain. In the initial growth stage of the electric field inside the cavity, ${|E(\vec{k},t)|}^{2}$ of each ASE mode is extremely small. Therefore, Eq. 4 can be approximated as

E(k→,t+τ)~exp(g0(∣Esol(k→)∣2/Isat1+∣Esol(k→)∣2/Isat))F−1[M(x→)F[E(k→,t)]] (5)

Under this approximation, the round trip mapping is linear. The fastest growing mode in this stage is hence the eigenmode of the linear mapping with the highest eigenvalue. When$I_{\text{sat}}>>{|E_{\text{sol}}(\vec{k})|}^{2}$, Eq. 5 is, to a good approximation, a projection on the compact support. Hence, all modes within the support grow exponentially faster than other modes. Thus, the solutions of the phase retrieval problem are both the fastest growing modes in the initial stage and the stable lasing modes. Moreover, since the phase retrieval problem has a unique solution, it assures that $E_{\text{sol}}(\vec{k})$ would be the only stable lasing mode from all the modes with a certain compact support. Thus, if the solution is present in the ASE, it is expected to be the lasing configuration of the system.

Unfortunately, there might be additional stable lasing modes in the nonlinear cavity that can lead to a wrong solution. Out of the possible stable lasing modes, which one is chosen by the system as the actual lasing mode? Before the lasing transition, the gain operates in the incoherent ASE regime, where the number of different phase configurations is very large, as quantified below. Each of these configurations evolves according to Eq. 4. Out of all these time-evolving phase configurations, the one with the highest energy (smallest loss) wins the mode competition over the limited gain. Therefore, the larger the number of initial independent configurations, the higher the probability of the system to find the correct solution, which is the unique stable configuration with no losses on the compact support mask.

Let us therefore evaluate the number of different ASE phase realizations inside the cavity. This number quantifies the independent parallel “computations” running simultaneously inside the cavity. These phase realizations are first amplified independently, until the gain medium approaches saturation. The nonlinear mode competition leads to the selection of the optimal configuration. A phase configuration is defined by a set of N phases, one for each of the N spatial modes in the cavity (i.e., the phase at each pixel on the SLM). For ASE, each spatial mode has a coherence length dictated by the bandwidth. The length of the cavity divided by this coherence length therefore dictates the number of different phases in each spatial mode in the cavity, denoted by *K*, the number of longitudinal modes. However, in ASE, the different spatial modes are independent. For simplicity, we model the phase of each spatial mode to evolve as a Poisson process with an average time between phase changes corresponding to the coherence length. Under this assumption, for *N* independent spatial modes, the rate at which the phase configuration changes is *N* times larger than that of a single mode. Therefore, the overall number of different phase realizations in the cavity is the number of longitudinal modes *K* times the number of spatial modes *N*, namely, *KN*.

In practice, our laser does not lase in a single longitudinal mode, although the number of modes decreases by a factor of 10 (*39*). For Nd–yttrium-aluminum-garnet (YAG) around 1064 nm, the ASE coherence length is about 2 mm; hence, in our 5-m cavity, there are about 2500 independent longitudinal modes. We estimate the number of spatial modes by the number of pixels in the SLM to be ~10^6^. Therefore, the overall number of different initial conditions is ~10^9^, and the number of final realizations in the cavity is ~10^2^.

## CONCLUSION

We presented an optical system for rapid phase retrieval, using a novel DDCL. The computation time is bounded by 100 ns, orders of magnitude faster than conventional computation systems (see, for comparison, reconstruction time with the RAAR algorithm in section S4). Note, however, that setting the scattered intensity distribution as the input on the SLM could take at least a few milliseconds. A direct approach, which uses the scattered light from the unknown object as an on-axis structured pump, could substantially speed up the process.

Several modifications to our DDCL system can potentially improve the performance. These include overcoming phase aberrations by adaptive calibration of the intracavity SLM (*40*), improving the stability and repeatability of pumping by resorting to diode pumping instead of flash light pumping, pump ramping in time to improve conversion to the ground state (*23*, *25*), introducing coupling between longitudinal cavity modes by either using an intracavity saturable absorber or measuring all different realizations inside the cavity and choosing the optimal one, and increasing the number of independent parallel realizations using a longer cavity and different gain medium with a broader spectrum of amplification (shorter correlation time in the ASE).

We believe that, in addition to rapidly finding solutions to phase retrieval problems, our DDCL systems can be exploited for solving a variety of problems that occur in many fields, e.g., resolving imagery after propagation through scattering media (*8*).

## MATERIALS AND METHODS

In our experiments, we actually used a reflective phase-only SLM, rather than a transmissive SLM, as depicted in the experimental arrangement shown in Fig. 1. The reflective SLM had a relatively high light efficiency and a high damage threshold. Accordingly, the laser arrangement was modified to retain the same operation functionality while accommodating the reflective SLM. The detailed experimental arrangement that includes the reflective SLM is schematically presented in fig. S1, along with an explanation on how a phase-only SLM together with the intracavity aperture can control the amplitude transmittance of each effective pixel in the SLM (*30*).

In our experimental arrangement, the laser gain medium was a 1.1% doped Nd-YAG rod of 10-mm diameter and 11-cm length. For quasi-CW operation, the gain medium was pumped high above threshold by a 100-μs pulsed xenon flash lamp operating at 1500 V and a repetition rate of 1 Hz to avoid thermal lensing. Each 4*f* telescope consists of two plano-convex lenses, with diameters of 50.8 mm and focal lengths of *f*_1_ = 750 mm and *f*_2_ = 500 mm at the lasing wavelength of 1064 nm. The SLM was Hamamatsu [liquid crystal on silicon (LCOS)–SLM X13138-03] with a high reflectivity of about 98% at 1064-nm wavelength, an area of 15.9 mm by 12.8 mm, 1272 × 1024 resolution, 12.5-μm pixel size, and a high damage threshold. Compact support aperture diameters ranged between 2 and 10 mm, depending on the object size. For Q-switch operation, a Pockels cell was incorporated into a linear degenerate cavity laser of the same gain and pump as the quasi-CW operation, and the focal lengths of two lenses in the telescope were 250 mm. Additional details are presented in section S2.

## Supplementary Material

http://advances.sciencemag.org/cgi/content/full/5/10/eaax4530/DC1

Download PDF

Rapid laser solver for the phase retrieval problem

## SUPPLEMENTARY MATERIALS

Supplementary material for this article is available at http://advances.sciencemag.org/cgi/content/full/5/10/eaax4530/DC1 Section S1. Detailed experimental arrangement Section S2. Convergence time to reach a solution Section S3. Simulation results Section S4. Runtime comparison to the RAAR phase retrieval algorithm Section S5. Phase measurement and reconstruction Fig. S1. Detailed experimental digital ring degenerate cavity laser arrangement. Fig. S2. Experimental arrangement with corresponding results for demonstrating rapid solutions. Fig. S3. Representative simulation results of inverse problem solutions with a DDCL. Fig. S4. Representative simulation results with phase aberrations. Fig. S5. Reconstruction fidelity in the RAAR algorithm. Fig. S6. Phase measurement methods. References (*41*–*44*)
