# Supplementary material for: Rapid laser solver for the phase retrieval problem
Source: Sci Adv. 2019 Oct 4;5(10):eaax4530. doi: 10.1126/sciadv.aax4530 (PMC6777974; doi:10.1126/sciadv.aax4530)
Supplement: http://advances.sciencemag.org/cgi/content/full/5/10/eaax4530/DC1 [file supp_5_10_eaax4530__index.html]

Science Advances | Science AdvancesAAASSearchScience AdvancesMenu

## Supplementary Materials

**This PDF file includes:**

- Section S1. Detailed experimental arrangement
- Section S2. Convergence time to reach a solution
- Section S3. Simulation results
- Section S4. Runtime comparison to the RAAR phase retrieval algorithm
- Section S5. Phase measurement and reconstruction
- Fig. S1. Detailed experimental digital ring degenerate cavity laser arrangement.
- Fig. S2. Experimental arrangement with corresponding results for demonstrating rapid solutions.
- Fig. S3. Representative simulation results of inverse problem solutions with a DDCL.
- Fig. S4. Representative simulation results with phase aberrations.
- Fig. S5. Reconstruction fidelity in the RAAR algorithm.
- Fig. S6. Phase measurement methods.
- References (*41*–*44*)

Download PDF

**Files in this Data Supplement:**

- Adobe PDF - aax4530\_SM.pdf
