# Supplementary material for: Rapid laser solver for the phase retrieval problem
Source: Sci Adv. 2019 Oct 4;5(10):eaax4530. doi: 10.1126/sciadv.aax4530 (PMC6777974; doi:10.1126/sciadv.aax4530)
Supplement: Download PDF [file aax4530_SM.pdf]

Science Advances
AAAS

advances.sciencemag.org/cgi/content/full/5/10/eaax4530/DC1

Supplementary Materials for

Rapid laser solver for the phase retrieval problem

C. Tradonsky*, I. Gershenzon, V. Pal, R. Chriki, A. A. Friesem, O. Raz, N. Davidson

*Corresponding author. Email: chene.tradonsky@weizmann.ac.il

Published 4 October 2019, *Sci. Adv.* **5**, eaax4530 (2019)
DOI: 10.1126/sciadv.aax4530

This PDF file includes:

Section S1. Detailed experimental arrangement
Section S2. Convergence time to reach a solution
Section S3. Simulation results
Section S4. Runtime comparison to the RAAR phase retrieval algorithm
Section S5. Phase measurement and reconstruction
Fig. S1. Detailed experimental digital ring degenerate cavity laser arrangement.
Fig. S2. Experimental arrangement with corresponding results for demonstrating rapid solutions.
Fig. S3. Representative simulation results of inverse problem solutions with a DDCL.
Fig. S4. Representative simulation results with phase aberrations.
Fig. S5. Reconstruction fidelity in the RAAR algorithm.
Fig. S6. Phase measurement methods.
References (*41–44*)

Supplementary Materials

Section S1. Detailed experimental arrangement

The detailed experimental arrangement of the digital degenerate cavity laser (DDCL) is schematically presented in fig. S1. It consists of a ring degenerate cavity laser that includes a gain medium, two 4f telescopes with one common lens, a reflective phase only spatial light modulator (SLM), an intra-cavity aperture, two retroreflectors and pentaprism-like 90° reflector (all from high reflectivity mirrors), two polarizing beam splitters (PBS), two half-wave plates ($\lambda/2$) and a Faraday rotator.

The operation of the detailed arrangement is essentially the same as the basic arrangement presented in Fig. 1. Each of the two 4f telescopes has one lens f_1 and a common lens f_2. The first telescope images the field distribution at the center of the gain medium onto the SLM where the reflectivity of each effective pixel (*30*) is controlled. The second telescope which contains an intra-cavity aperture, images the field distribution at the SLM back onto the gain medium. Such field distribution is determined by the size and shape of the intra-cavity aperture (compact support). Since our SLM operates on axis and by reflection on horizontal polarized light, half of the ring degenerate cavity was designed as a twisted-mode (*41*) linear degenerate cavity (*31*) and the other half as regular ring cavity laser (*31*). The two halves are connected by PBS_1, which separates the two counter-propagating beams to two different cross-polarized paths. A large aperture Faraday rotator together with a half-wave plate (HWP) at 22.5° and another PBS_2 (which also serves as ~5% output coupler) enforce unidirectional operation of the ring cavity. A 90° reflector flips left and right areas of the beam. The left retroreflector can compensate for free propagation diffraction in the cavity. The right retroreflector can compensate for spherical phase aberrations in the cavity. A second HWP at 45° rotates the polarization from vertical to horizontal to pass through PBS_1. The detection arrangement includes a CMOS camera and lenses so both the reconstructed object and the scattered intensity distributions can be detected.

The local reflectivity magnitude of the SLM is determined by local phase differences between adjacent pixels and affects the amount of light diffracted out of the laser cavity. The local reflectivity phase is determined by the local average phase of the adjacent pixels (*30*). For example, adjacent pixels with phases of [0, 0] will result in high reflectivity and 0 phase, whereas adjacent pixels with phases of [0, π] will result in no reflectivity and $\pi/2$ phase. The reflectivity pattern can be used to form any desired intensity distribution and the phase distribution can be used to overcome aberrations in the cavity and increase the laser degeneracy. For the phase retrieval problem, the SLM local reflectivity should ideally be set by the function of the input Fourier intensity distribution and cavity parameters, as given by Eq. (3). However, the direct application of Eq. (3) in the experimental system is not possible due to spatial inhomogeneities, caused by technical imperfections. The imperfections result from a non-uniform spatial pump profile and a non-uniform spatial loss profile caused by cavity phase aberrations. To compensate for these inhomogeneities, we set the SLM local reflectivity using an iterative computer-mediated feedback loop. This feedback loop is designed to stabilize the lasing intensity distribution at the SLM plane to coincide with the input Fourier intensity at each point. Specifically, we employed a proportional control scheme (*42*) where the variable is the lasing

intensity at the SLM plane and the target value is given by the input Fourier intensity. Formally, the reflectivity of the ith SLM pixel after n iterations, $R_i^{(\mathrm{n})}$, is given by $R_i^{(\mathrm{n})} = R_i^{(\mathrm{n\text{-}1})} + p\left(\left|E_{\mathrm{sol}}(\vec{k_i})\right|^2 - I_{\mathrm{meas},i}^{(\mathrm{n\text{-}1})}\right)$ where p is a feedback parameter, $I_{\mathrm{meas},i}^{(\mathrm{n\text{-}1})}$ is the measured lasing intensity at the ith SLM pixel after $n-1$ iterations, and $\left|E_{\mathrm{sol}}(\vec{k_i})\right|^2$ is the input Fourier intensity at the ith pixel. This procedure provides equivalent results to those that would be obtained by using Eq. 3 directly, whereby the required lasing intensity distribution at the SLM plane, i.e. the input Fourier intensity distribution, is the same. Note that no relative phase is imposed between adjacent pixels so the lasing frequencies of all the pixels are identical, leading to an interference pattern that forms the reconstructed object at the compact support plane, i.e. the solution to the phase retrieval problem.

Section S2. Convergence time to reach a solution

In order to determine the time it takes the laser to solve the phase retrieval problem, we performed experiments with Q-switched laser to obtain a narrow pulse. For this purpose, we resorted to a Q-switched linear DCL, schematically presented in fig. S2A. The Q-switched linear DCL comprises two lenses in a 4f telescope configuration, a Pockels cell and two intra-cavity amplitude masks. The first mask was placed near the rear mirror and served to enforce a specific input scattered intensity distribution according to the phase retrieval problem. Here we used a metallic binary amplitude mask instead of an SLM, due to high peak power of Q-switched operation of the laser and the limited damage threshold of the SLM. The second mask was placed between the two lenses and served as the compact support.

The results are presented in figs. S2B-F. Figure S2B shows the pulse intensity profile of the laser was about 100ns. Figures S2C-F show the intensity distributions at the mask (representing the scattered intensity distribution from the unknown object) and the intensity distributions of the reconstructed object at the compact support plane. Figure S2C and fig. S2D show the results at quasi-CW lasing (no Q-switching), and fig. S2E and fig. S2F show the results at Q-switched lasing operation with pulse duration of 100ns. As evident, the short duration of the pulse does not affect the quality of the reconstructed object. Although we estimate the convergence time to reach a solution would be significantly shorter than 100ns, we set this value as the upper bound.

Section S3. Simulation results

In order to support our experimental results, we performed some basic simulations. Specifically, we simulated the field distribution of the transverse mode inside the laser cavity (representing the reconstructed object) in the arrangement shown in Fig. 1, by resorting to a modified Gerchberg-Saxton (GS) iterative algorithm. In the simulation, we included the phase only SLM, laser gain medium and the aperture shaped as compact support of the object. We started with an initial guess of a random field distribution at the SLM plane, and then resorted to the iterative algorithm according to Eq. 3. In each iteration, the field of the next round trip was calculated from the current one.

Representative simulation results are presented in fig. S3. Figure S3A shows an image of the actual scattering object. Figure S3B shows the simulated diffracted intensity distribution that served as the input pattern inside the laser cavity. Figure S3C shows the reconstructed intensity distribution of the object after 100 iterations inside the laser cavity. The compact support shape

in this example was the outer boundary of the object, while the details inside were reconstructed by the algorithm. Note that the simulations only deal with a single realization, out of the thousands parallel realizations that run in the cavity and compete on the gain.

We also investigated the effect of phase aberrations, spherical and others that are caused by the SLM, on the resolution of the reconstructed object. This was done by incorporating phase aberrations into the modified GS algorithm simulations. The results are presented in fig. S4. Figures S4A, B and C show the simulation results for a low-resolution object and without any aberrations in our system. Figure S4A shows the intensity distribution of the actual object, fig. S4B the corresponding simulated intensity distribution of the diffraction pattern inside the laser cavity and fig. S4C the intensity distribution of the reconstructed object after 100 iterations inside the laser cavity. Figures S4D, E and F show the simulation results after adding typical aberrations from the SLM manufacturer calibration file (contains phase corrections to make SLM surface flat). Figure S4D shows the typical phase aberrations distribution caused by the SLM, fig. S4E the corresponding simulated intensity distribution of the input diffraction pattern inside the cavity, and fig. S4F the intensity distribution of the reconstructed object after 200 iterations inside the laser cavity. Figures. S4G, H and I show the simulation results after subtracting spherical aberrations but retaining the others. Figure S4G shows the improved phase aberrations distribution, fig. S4H the corresponding simulated intensity distribution of the input diffraction pattern inside the cavity and fig. S4I the intensity distribution of the reconstructed object after 200 iterations inside the laser cavity. These results indicate that phase aberrations strongly affect the ability of the system to successfully reach the correct solution and reconstructed object that accurately matches the actual object. For example, fig. S4F shows a poor reconstructed object when all phase aberrations of the SLM are taken into account. There is an improvement when the spherical aberrations are removed, as shown in fig. S4I. This correction of the phase aberrations is done using a linear shift (right or left) of the retroreflector 1 in fig. S1. As evident, the resolution of the reconstructed object is similar to that of our experimental results. In order to confirm that our approach can potentially lead to perfect reconstructed objects, we performed simulations using an object with fine features and sharp edges (high spatial frequencies) and assumed no phase aberrations (perfect cavity). The results are shown in figs. S4J, K and L. Figure S4J shows the intensity distribution of the actual object, fig. S4K the simulated intensity distribution of the diffraction pattern inside the cavity, and fig. S4I the intensity distribution of the high-resolution reconstructed object after 200 iterations inside the laser cavity. These results clearly verify the efficacy of our approach.

Section S4. Runtime comparison to the RAAR phase retrieval algorithm

In order to compare the DDCL runtime to that of a state-of-the-art phase retrieval algorithm, we used the open source PRwSC package. To ensure that we compare problems with the same computational difficulty, we applied the RAAR algorithm (12) on the object from the lowest row in Fig. 3. The input to the RAAR algorithm was the diffraction pattern amplitudes, identical in resolution to the SLM input of the DDCL, and a compact support identical to the mask used in the cavity (dashed white circle with wedge in the center of the lowest row in Fig. 3B). The feedback parameter of the RAAR algorithm, commonly denoted by β, was chosen to be β=0.83: this value gave the fastest convergence in this specific problem. The initial condition for the algorithm was a random phase realization, where the phase of each diffraction pixel was chosen randomly from a uniform distribution in the range [0:2π]. To ensure that there is no strong

dependence on the chosen initial phases, we repeated the reconstruction procedure 1000 times, initiating the algorithm with a different random phase realization each time.

A representative example of the reconstruction fidelity as a function of the iteration number is shown in fig. S5. Typically, the algorithm converged to the correct solution after less than 150 iterations. A histogram of the reconstruction fidelity is shown in fig. S5. The median of the reconstruction fidelity after 150 iterations is 0.95. In 806 out of 1000 trials, the reconstruction fidelity was above 0.8 after 150 iterations. Thus, the RAAR failed to converge to the correct solution (fidelity above 0.8) for ~20% of the realizations. This remain the case even when 1000 RAAR iterations are used. Moreover, the convergence curve is often non monotonic, namely there are local minima on which even the RAAR algorithm stagnates. This agrees with the common belief that objects that have non-uniform phase distribution are more difficult to reconstruct. The averaged reconstruction time of 150 iterations on a 4.2 GHz, Intel i7 quad-core processor is 1.05 seconds. In conclusion, on a modern computer the RAAR is able to solve this specific problem in about 1 second.

Section S5. Phase measurement and reconstruction

To demonstrate that our system indeed solves the phase retrieval problem by directly measuring the reconstructed phase, we placed a Mach-Zehnder interferometer following the output coupler of the laser cavity *(43, 44)*. These results are presented on fig. S6. The original object and object's Fourier intensity and phase are shown in column A of fig. S6. The measured Fourier phase superimposed on the input Fourier intensity and corresponding object intensity and phase are shown in column B of fig. S6. of column B, we present the measured phase in the Fourier plane, superimposed on the input Fourier intensity.

The measured phase is visually in good agreement with the original Fourier intensity and phases of the object. To estimate quantitatively the quality of the measured phases, we evaluated a normalized square error between the Fourier fields in the following manner

$$\text{Error} = \frac{\left\|E_{\text{meas}}(k) - E_{\text{orig}}(k)\right\|_2^2}{\left\|E_{\text{orig}}(k)\right\|_2^2} \quad \text{(S1)}$$

where $E_{\text{meas}}(k)$ and $E_{\text{orig}}(k)$ are the measured and original complex fields of the object. The error of the measured Fourier field is 0.53. The intensity and phase at the object plane can now be obtained by an inverse Fourier transform of the Fourier field and are presented in the lower panel of column B. The object field visually captures the object intensity while the object phase reconstruction quality is relatively poor. We believe this to be due to residual linear phase in the object plane as well as aberrations in the phase measurement apparatus.

As discussed in the main text, the measurement of the reconstructed object intensity pattern is theoretically sufficient to obtain the full phase information. We show in fig. S6 column C that a few iterations of a simple GS algorithm are sufficient for the reconstruction of the phase. The input Fourier intensity and the measured object intensity are fed into a GS algorithm as an initial guess (with a random phase distribution). The obtained Fourier distribution C and original object phase A are in agreement, with an error of 0.33. The number of iterations required for convergence to this solution was 24.

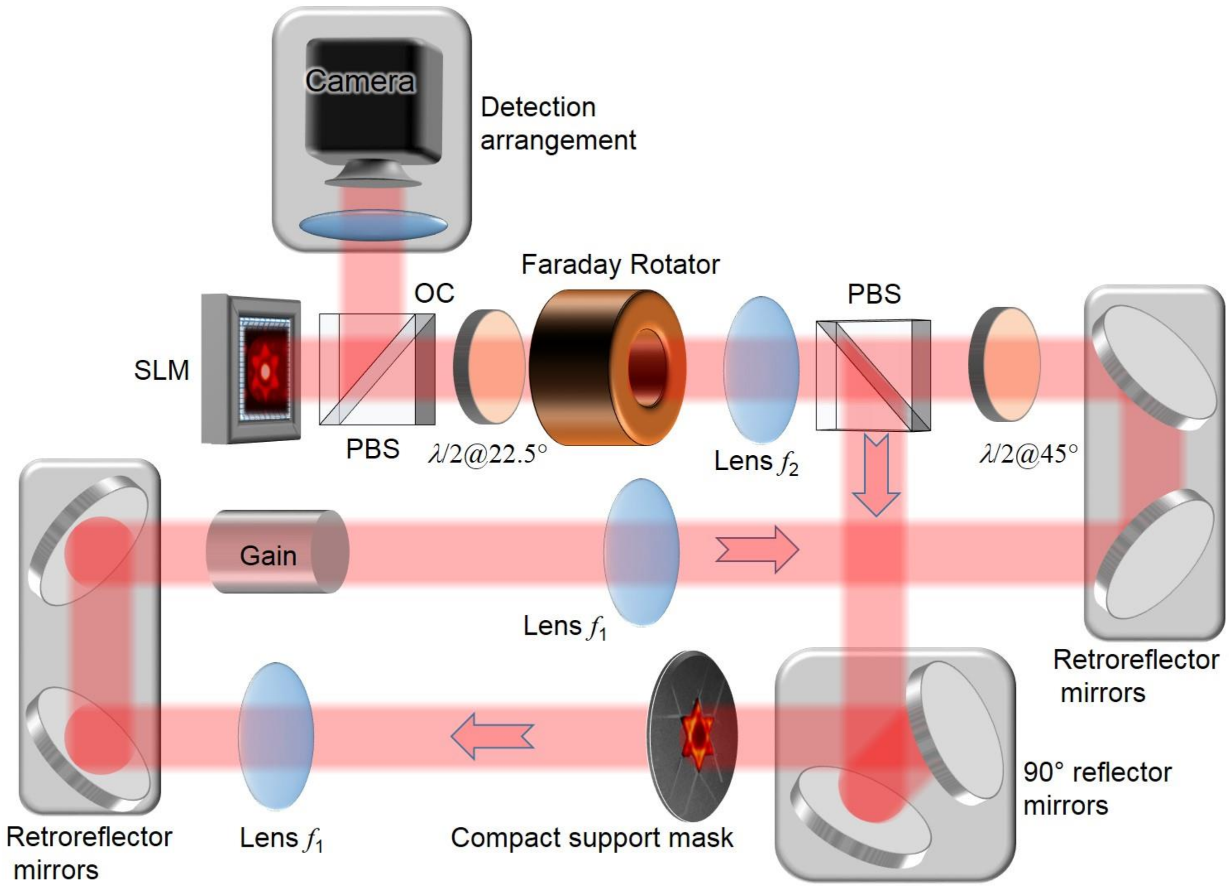

Fig. S1. Detailed experimental digital ring degenerate cavity laser arrangement. SLM - spatial light modulator; PBS - polarizing beam splitter; λ/2@22.5° - half-wave plate at 22.5° angular orientation; λ/2@45° - half-wave plate at 45° angular orientation; compact support mask - intra-cavity aperture at the Fourier plane; OC–output coupler.

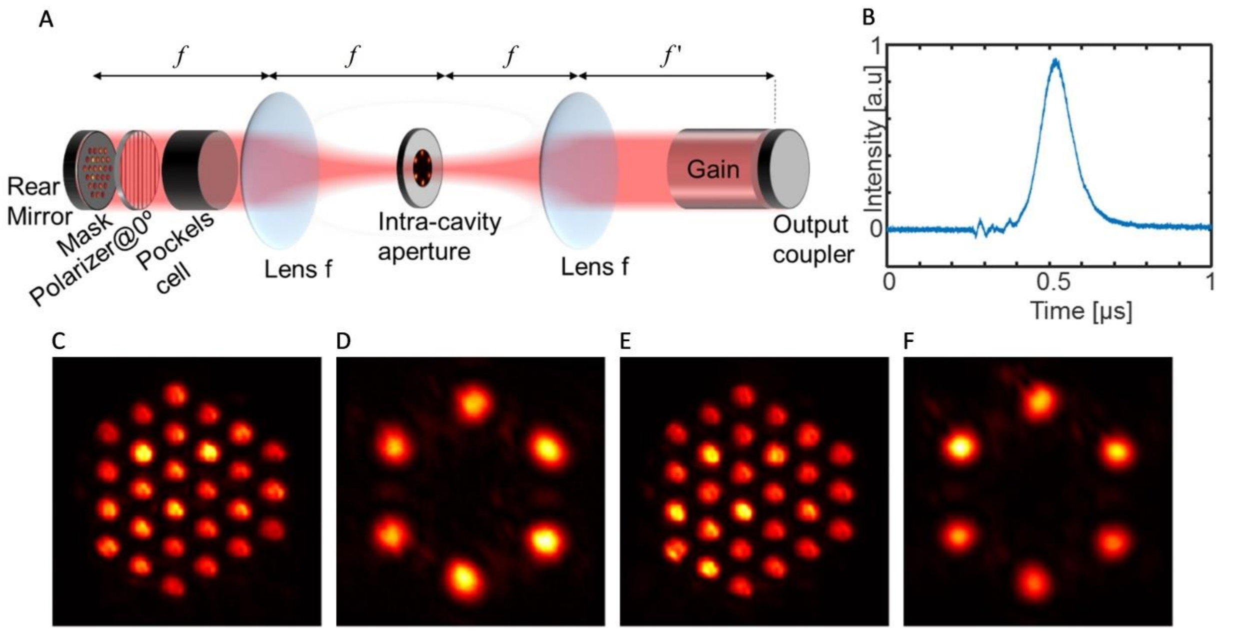

Fig. S2. Experimental arrangement with corresponding results for demonstrating rapid solutions. A - A linear Q-switched DCL with Pockels cell and two intra-cavity masks. B - Laser intensity as a function of time. The duration of the lasing process (convergence to a solution) is limited to about 100ns by incorporating a Pockels cell into the laser cavity. C and D - Scattered and reconstructed object intensity distributions, at quasi-continuous lasing operations (without Q switch). E and F - Scattered and reconstructed object intensity distributions, at Q-switched lasing operations with pulse duration of 100ns.

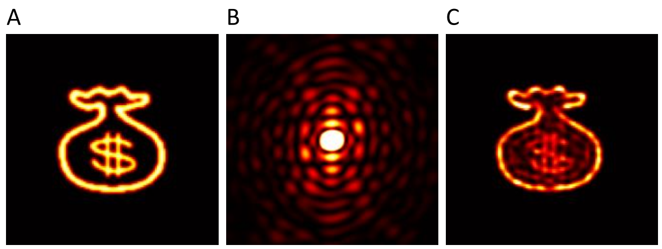

Fig. S3. Representative simulation results of inverse problem solutions with a DDCL. A - Image of the actual scattering object. B - Simulated intensity distribution of the diffraction pattern inside the cavity. C - Intensity distribution of the reconstructed object after 100 iterations inside the cavity.

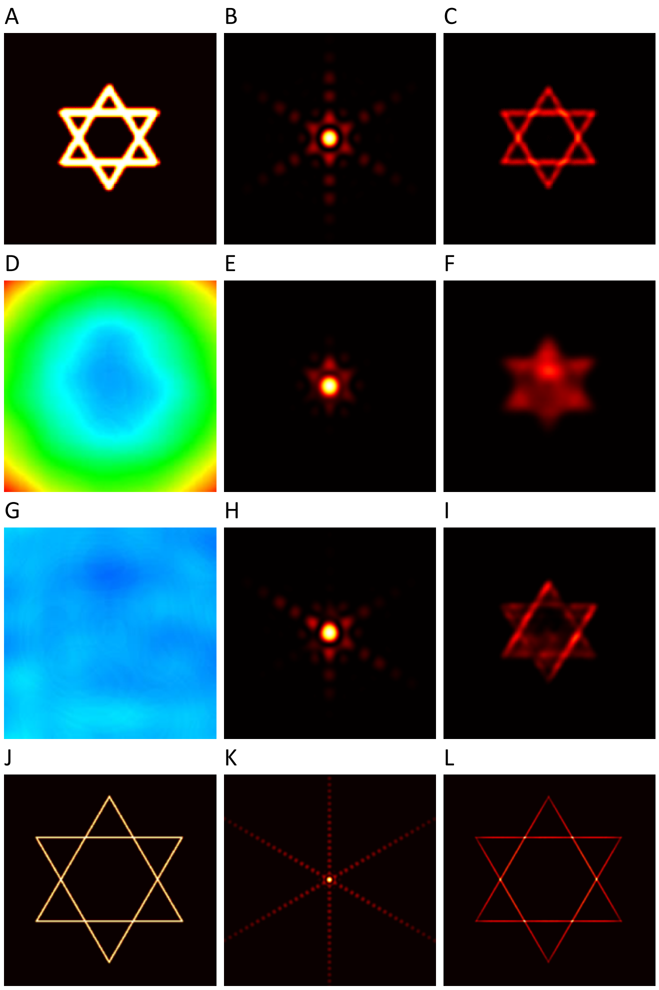

Fig. S4. Representative simulation results with phase aberrations. A - Image of the actual scattering object at low-resolution (LR). B - Simulated intensity distribution of the LR diffraction pattern inside the cavity. C - Intensity distribution of the reconstructed object after 100 iterations inside the cavity. D and G - Typical phase aberration of SLM (from the calibration file of the SLM) and after subtraction of defocusing aberration. E and H - Simulated intensity distributions of the LR diffraction patterns inside the cavity. F and I - Intensity distributions of the reconstructed object after 200 iterations inside the cavity with phase aberrations given in D and G. J - Image of the actual scattering object at high-resolution (HR). K - Simulated intensity distribution of the HR diffraction pattern inside the cavity. L - Intensity distribution of the reconstructed object after 200 iterations inside the cavity.

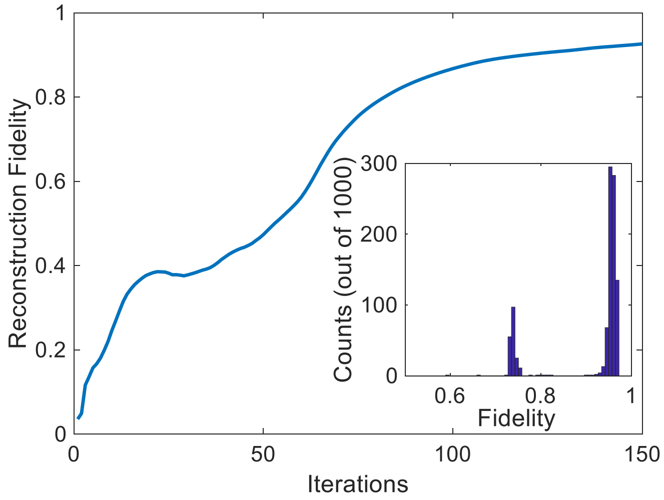

Fig. S5. Reconstruction fidelity in the RAAR algorithm. A typical convergence curve of the RAAR algorithm in the phase problem at the lowest panel of Fig. 3. The reconstruction fidelity is defined in Eq. 1. After 150 iterations (about 1 second) the algorithm converges to a good solution. The inset shows a histogram of the reconstruction fidelity in 1000 trails, initiated at random phases.

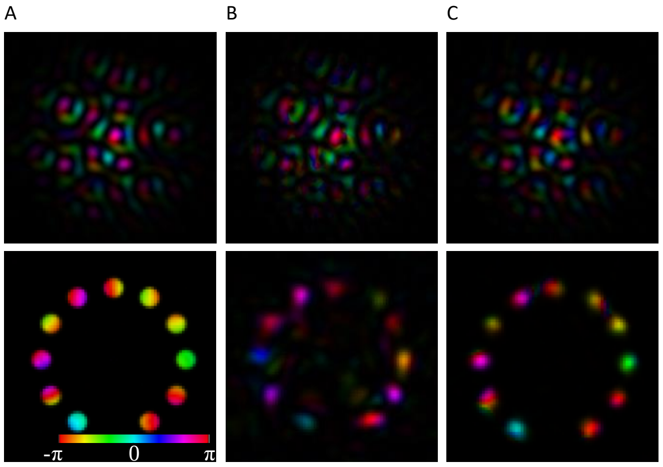

Fig. S6. Phase measurement methods. Each Column contains the Fourier intensity (brightness) and phase (hue) in the upper panel, and the object intensity and phase distributions in the lower panel. Column A - The original object. Column B - Upper panel – directly measured Fourier phase superimposed on the input Fourier intensity, lower panel – object intensity and phase obtained by inverse Fourier transform of the Fourier field. Column C - Upper panel – numerically obtained phase superimposed on input Fourier intensity, lower panel – numerically obtained phase superimposed on measured object intensity.
